# The discriminative role of angiopoietin-like protein-3 for metabolic syndrome in polycystic ovary syndrome

**DOI:** 10.1590/1806-9282.20251263

**Published:** 2026-05-11

**Authors:** Özgenur Ayvaz Karaca, Gulten Ozgen, Ozlem Dogan, Burcu Dincgez, Levent Ozgen

**Affiliations:** 1 Kestel State Hospital, Department of Obstetrics and Gynecology – Bursa, Turkey.; 2 University of Health Sciences, Bursa Yuksek Ihtisas Research and Training Hospital, Department of Obstetrics and Gynecology – Bursa, Turkey.; 3 Ankara University, Faculty of Medicine, Department of Medical Biochemistry – Ankara, Turkey.

**Keywords:** Angiopoietin-like protein 3, Metabolic syndrome, Polycystic ovary syndrome

## Abstract

**OBJECTIVE:**

Patients with polycystic ovary syndrome face an increased risk of developing metabolic syndrome. Identifying biomarkers that can detect metabolic syndrome in polycystic ovary syndrome is essential, as it may enable physicians to implement preventive strategies aimed at reducing the risk of metabolic complications. The aim of this study was to assess angiopoietin-like protein-3 levels in polycystic ovary syndrome and to investigate the potential of angiopoietin-like protein-3 for discriminating metabolic syndrome in polycystic ovary syndrome.

**METHODS:**

A total of 50 women with polycystic ovary syndrome and 45 controls were included in this prospective study. Then, polycystic ovary syndrome patients were categorized into two subgroups: metabolic syndrome-positive (n=15) and metabolic syndrome-negative polycystic ovary syndrome (n=35). Age, parity, body mass index, blood pressure, waist circumference, Ferriman-Gallwey scores, hormone and lipid profiles, glucose, insulin, C-reactive protein, procalcitonin, and angiopoietin-like protein-3 levels were compared between groups.

**RESULTS:**

The median angiopoietin-like protein-3 level was significantly higher in polycystic ovary syndrome as compared to controls [41.19 (15.28–69.56) vs. 29.11 (9.85–66.25) ng/mL, p<0.001]. Similarly, angiopoietin-like protein-3 levels were higher in metabolic syndrome-positive polycystic ovary syndrome patients compared to those without metabolic syndrome (p=0.010). Angiopoietin-like protein-3 >32.5 ng/mL yielded a sensitivity of 76% and a specificity of 66.7% for predicting polycystic ovary syndrome (p<0.001, AUC=0.751), while angiopoietin-like protein-3 >44.54 ng/mL provided 68.8% sensitivity and 79.4% specificity for predicting metabolic syndrome in polycystic ovary syndrome (p=0.002, AUC=0.759). No correlation was detected between angiopoietin-like protein-3 and systolic blood pressure, body mass index, waist circumference, glucose, homeostatic model assessment insulin resistance, lipids, and inflammatory markers in polycystic ovary syndrome patients with metabolic syndrome.

**CONCLUSION:**

Angiopoietin-like protein-3 could discriminate metabolic syndrome in polycystic ovary syndrome patients. However, given that the correlation analysis did not find any association between Angiopoietin-like protein-3 and any etiopathogenesis-related characteristic, we think that further research is necessary to determine how it accomplishes this.

## INTRODUCTION

Polycystic ovary syndrome (PCOS) is the most prevalent endocrine disorder among women of reproductive age. Beyond immediate concerns like menstrual disturbances and infertility, PCOS carries long-term health risks including obesity, hypertension, dyslipidemia, and insulin resistance—all key features of metabolic syndrome (MS)^1^.

In recent decades, MS has emerged as a growing global health concern among the general population. It increases the risk of developing diabetes mellitus, cardiovascular diseases, and stroke. The key components of the syndrome include central obesity, high blood pressure, increased glucose and triglyceride levels, and decreased levels of high-density lipoprotein (HDL). PCOS patients are at a higher risk of developing MS. Although the exact cause of MS in women with PCOS remains unclear, insulin resistance and obesity are widely considered contributing factors^1,2^. Clinical guidelines recommend screening for MS in women diagnosed with PCOS^3,4^. Identifying biomarkers that can detect MS in the early stages of PCOS is essential, as it may enable physicians to implement preventive strategies aimed at reducing the risk of metabolic complications.

Angiopoietin-like protein-3 (ANGPTL-3) plays a key role in regulating lipid metabolism, acting as a potent inhibitor of lipoprotein lipase and endothelial lipase^5^. Considering these effects, ANGPTL-3 has been highlighted as one of the most promising emerging targets for cardiometabolic therapy^5-7^.

To the best of our knowledge, limited information is available regarding the relationship between ANGPTL-3 and PCOS. A previous study reported elevated ANGPTL-3 levels in PCOS patients and found an association with insulin resistance^7^. However, no studies to date have investigated the role of ANGPTL-3 in distinguishing PCOS patients with MS from those without MS. In the present study, we aimed to assess ANGPTL-3 levels in PCOS and to investigate the potential of ANGPTL-3 for discriminating MS in PCOS.

## METHODS

This prospective case–control study was conducted between December 2021 and July 2022. Ethical approval was obtained from the local ethics committee, and written informed consent was obtained.

### Study population

A total of 95 participants were enrolled, including 50 women with PCOS and 45 healthy controls. Exclusion criteria included the presence of hypothyroidism, hyperprolactinemia, or any condition that could cause menstrual irregularities or androgen excess, such as non-classical congenital adrenal hyperplasia or Cushing's syndrome. Participants with systemic conditions including diabetes, hypertension, autoimmune disorders, and cardiovascular, renal, or hepatic diseases were also excluded. The control group was composed of women between 18 and 35 years old with regular menstrual cycles and normal hormonal profiles. Additionally, patients with comorbidities, who used hormone therapy or any medications within the last 3 months, and who had evidence of hirsutism were excluded from the control group.

PCOS was diagnosed based on the Rotterdam criteria established by the American Society for Reproductive Medicine and the European Society for Human Reproduction and Embryology^3^. Subsequently, the patients with PCOS were categorized into two subgroups: MS-positive (n=15) and MS-negative (n=35). MS was diagnosed using a modified version of the National Cholesterol Education Program Adult Treatment Panel III criteria^1^.

Power analysis was performed, which revealed that the minimum patient number was 14 for both MS-positive and MS-negative cases, with 80% power to detect a 30% difference in cases with a value of 0.05.

Data on age, parity, body mass index (BMI), blood pressure, waist circumference (WC), and M-FGS were recorded.

### Biochemical measurements

Blood samples were collected during the early follicular phase in menstruating participants and on a random day for amenorrheic patients. Serum levels were analyzed for hormone and lipid profile, glucose, insulin, CRP, procalcitonin, and ANGPTL-3. ANGPTL-3 was measured using an enzyme-linked immunosorbent assay method with a commercial kit (Human ANGPTL-3 ELISA Kit, ELK Biotechnology, China). The inter-assay CV was 3.4% and intra-assay CV was 6%.

### Statistical analysis

The normality of data distribution was evaluated with Shapiro-Wilk test. For comparisons of continuous variables between two groups, the Student's t-test and Mann-Whitney U test were used. Categorical variables were analyzed using chi-square test or Fisher's exact test. Descriptive statistics were presented as mean±standard deviation, median (minimum–maximum), and frequency (percentage). The predictive value of ANGPTL-3 for PCOS and MS was evaluated with receiver operating characteristics (ROC) analysis. Spearman's correlation coefficient was used to determine the relationship between ANGPTL-3 and clinical parameters. MedCalc18 and SPSS22.0 were used for analysis, and p-value ≤0.05 was considered statistically significant.

## RESULTS

Table 1 summarizes the clinical characteristics and laboratory findings of the PCOS and control groups. In the PCOS group, WC, systolic blood pressure, M-FGS, LH, total testosterone, androstenedione, insulin levels, and HOMA-IR and ANGPTL-3 levels were elevated, whereas levels of HDL were reduced.

**Table 1:** The clinical characteristics and laboratory parameters of the polycystic ovary syndrome and control group.

	PCOS (n=50)	Control (n=45)	p-value
Age (years)	23.5 (18–35)	24 (19–35)	0.082
Parity (n)	1 (0–3)	0 (0–3)	0.577
BMI (kg/m²)	24.96 (17.1–40.23)	22.9 (18.3–36.7)	0.140
WC (cm)	80.13±10.17	75.26±13.01	**0.036**
Systolic blood pressure (mmHg)	118.0±8.3	113.7±8.6	**0.015**
Diastolic blood pressure (mmHg)	77 (63–87)	75 (62–95)	0.404
M-FGS	6 (0–16)	1 (0–9)	**<0.001**
FSH (mIU/mL)	5.28±1.77	5.39±1.8	0.770
LH (mIU/mL)	4.7 (0.7–17.8)	3.4 (0.9–8.5)	**0.006**
E2 (pg/mL)	44 (2.2–206)	41 (18–298)	0.809
Total testosterone (nmol/L)	45 (3.3–148.5)	36.4 (20.4–53.7)	**0.001**
DHEA-S (µg/dL)	245.8 (68.2–794.8)	296 (71.8–467.2)	**0.037**
Androstenedione (ng/mL)	1.34 (0.32–2.74)	0.87 (0.5–2.45)	**0.001**
Glucose (mg/dL)	87.2±12.1	87.4±11.7	0.929
Insulin (µU/mL)	10.5 (3.5–66.2)	7.9 (1.9–31.5)	**0.017**
HOMA-IR (mg/dL)	2.2 (0.7–17)	1.7 (0.4–8.7)	**0.034**
Triglyceride (mg/dL)	93.5 (37–298)	73 (17–284)	0.051
Total cholesterol (mg/dL)	163.11±31.61	154.95±22.27	0.116
LDL (mg/dL)	95.3±30.8	87.7±23.5	0.183
HDL (mg/dL)	51.3 (30.6–158)	60 (37.3–125.8)	**0.006**
CRP (mg/dL)	3.3 (3–8.9)	3.3 (2.9–29)	0.391
Procalcitonin (ng/mL)	0.01 (0–1.01)	0.01 (0–0.03)	0.763
ANGPTL-3 (ng/mL)	41.19 (15.28–69.56)	29.11 (9.85–66.25)	**<0.001**

ANGPTL-3: angiopoietin-like protein-3; BMI: body mass index; CRP: C-reactive protein; DHEA-S: dehydroepiandrosterone sulfate; E2: estradiol; FSH: follicle-stimulating hormone; HDL: high-density lipoprotein; HOMA-IR: homeostatic model assessment insulin resistance; LDL: low-density lipoprotein; LH: luteinizing hormone; M-FGS: modified Ferriman-Gallwey score; WC: waist circumference; PCOS: polycystic ovary syndrome. Bold values indicate statistically significant results (p<0.05).

Table 2 displays the clinical characteristics and laboratory parameters of PCOS patients with and without MS. BMI, WC, M-FGS, and triglyceride levels were elevated, while HDL was reduced in PCOS with MS. ANGPTL-3 levels were elevated in PCOS with MS compared to those without MS (p=0.010).

**Table 2:** The clinical characteristics and laboratory parameters of metabolic syndrome-positive and -negative polycystic ovary syndrome patients.

	MS-positive PCOS (n=15)	MS-negative PCOS (n=35)	p-value
Age (years)	25 (18–35)	23 (18–35)	0.177
Parity (n)	1 (0–3)	1 (0–2)	0.745
BMI (kg/m²)	31.1±5.16	23.64±4.34	**<0.001**
WC (cm)	96 (79–114)	78 (62–103)	**<0.001**
Systolic blood pressure (mmHg)	119.5±9.21	117.4±7.99	0.421
Diastolic blood pressure (mmHg)	78 (63–87)	75 (67–85)	0.633
M-FGS	8 (0–15)	6 (0–16)	**0.041**
FSH (mIU/mL)	5.85±1.95	5.04±1.66	0.139
LH (mIU/mL)	5.44 (2.75–17.8)	4.67 (0.74–14.3)	0.295
E2 (pg/mL)	49 (25–69)	41 (2.2–206)	0.505
Total testosterone (nmol/L)	49.1 (24.6–81.5)	42.3 (21.6–148.5)	0.159
DHEA-S (µg/dL)	230.7 (79–647)	266.6 (68.2–794.8)	0.874
Androstenedione (ng/mL)	1.46±0.53	1.33±0.58	0.445
Glucose (mg/dL)	93 (67–104)	86 (51–110)	0.439
Insulin (µU/mL)	13.2 (3.5–66.2)	9.6 (3.7–37.4)	0.074
HOMA-IR (mg/dL)	2.4 (0.70–17)	2.1 (0.8–8.8)	0.132
Triglyceride (mg/dL)	159 (53–298)	83 (37–174)	**<0.001**
Total cholesterol (mg/dL)	176.31±21.09	159.35±11.61	0.841
LDL (mg/dL)	96.4±35.8	94.8±28.9	0.874
HDL (mg/dL)	44 (33.5–158)	55 (30.6–81.4)	**0.001**
CRP (mg/dL)	3.3 (3–8.9)	3.3 (3–5.9)	0.243
Procalcitonin (ng/mL)	0.01 (0–1.01)	0.01 (0–0.01)	0.644
ANGPTL-3 (ng/mL)	48.8±14.4	37.3±9.18	**0.010**

ANGPTL-3: angiopoietin-like protein-3; BMI: body mass index; CRP: C-reactive protein; DHEA-S: dehydroepiandrosterone sulfate; E2: estradiol; FSH: follicle-stimulating hormone; HDL: high-density lipoprotein; HOMA-IR: homeostatic model assessment insulin resistance; LDL: low-density lipoprotein; LH: luteinizing hormone; M-FGS: modified Ferriman-Gallwey score; WC: waist circumference; MS: metabolic syndrome; PCOS: polycystic ovary syndrome. Bold values indicate statistically significant results (p<0.05).

**Figure 1:**
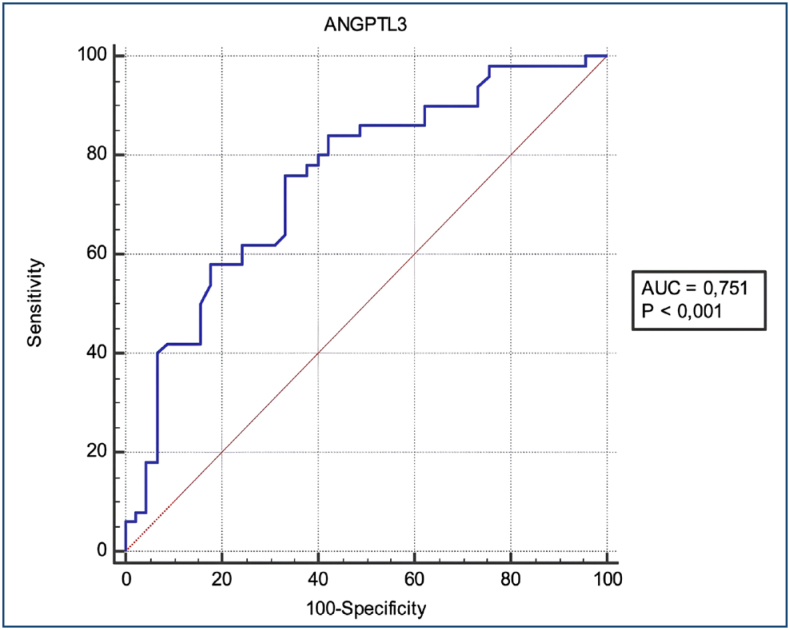
Receiver operating characteristics analysis evaluating the potential of angiopoietin-like protein-3 as a predictive marker for polycystic ovary syndrome.

The potential of ANGPTL-3 as a predictive marker for PCOS was assessed using ROC analysis (Figure 1). Based on this analysis, a cut-off value of 32.5 ng/mL yielded a sensitivity of 76% and a specificity of 66.7% for predicting PCOS (p<0.001, AUC=0.751).

**Figure 2:**
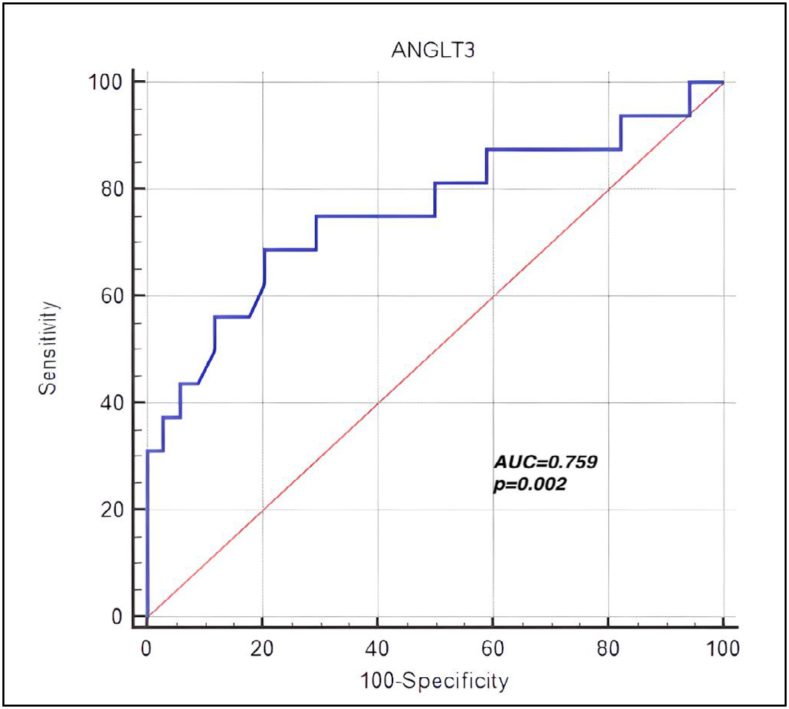
Receiver operating characteristics analysis evaluating the ability of angiopoietin-like protein-3 to predict metabolic syndrome in polycystic ovary syndrome.

The ROC analysis evaluating the ability of ANGPTL-3 to predict MS in PCOS is shown in Figure 2. The analysis identified a cut-off value of 44.54 ng/mL, providing 68.8% sensitivity and 79.4% specificity for predicting MS in PCOS patients (p=0.002, AUC=0.759). This predictive role was compared with HOMA-IR, and no significant difference was found between ANGPTL-3 and HOMA-IR (AUC=0.759 vs. AUC=0.635, p=0.341).

In PCOS patients, ANGPTL-3 levels showed no correlation with systolic blood pressure (p=0.274), WC (p=0.081), fasting glucose (p=0.271), HOMA-IR (p=0.759), and total testosterone (p=0.277). ANGPTL-3 was positively correlated with BMI (r=0.365, p=0.009), triglyceride (r=0.359, p=0.011), CRP (r=0.387, p=0.023) and negatively correlated with HDL (r=-0.315, p=0.026).

In PCOS patients with MS, ANGPTL-3 levels showed no significant correlation with systolic blood pressure (p=0.250), BMI (p=0.231), WC (p=0.819), glucose (p=0.829), HOMA-IR (p=0.874), total testosterone (p=1.000), triglyceride (p=0.487), HDL (p=0.965), CRP (p=0.442), and procalcitonin (p=0.367).

## DISCUSSION

PCOS frequently presents with obesity, insulin resistance, and dyslipidemia^1,7^. Given the strong link between MS, insulin resistance, and inflammation, MS is commonly observed in patients with PCOS^1^. Due to its serious long-term health implications, numerous studies are focused on identifying biomarkers that can predict the presence of MS in PCOS.

The angiopoietin-like protein family participates in many biological processes, including lipid and glucose metabolism, inflammation, and tumor progression^7,8^. Elevated ANGPTL-3 levels have been found to be associated with dyslipidemia, cardiovascular disease, insulin resistance, hyperlipidemia-related proteinuria, and severe non-alcoholic fatty liver disease, while loss-of-function mutations in ANGPTL-3 have been linked to familial combined hypobetalipoproteinemia^6-10^. Furthermore, ANGPTL-3 levels have shown a positive correlation with glucose, insulin, and HOMA-IR levels in diabetes and obesity^11^. In genome-wide association studies, ANGPTL-3 variants have been found to be associated with dyslipidemia, diabetes, and obesity^12-14^. In contrast, Shen et al. showed no significant relationship of ANGPTL-3 SNP rs1748195 with the risk of dyslipidemia in Chinese children^15^. A study assessing the association of the rs1748195 variant in ANGPTL-3 and the risk of diabetes demonstrated that individuals with a GG genotype compared to a CG genotype had a lower risk of diabetes in patients with cardiovascular disease and a low risk of obesity in the control group^16^.

To date, only one study has evaluated ANGPTL-3 in PCOS, reporting elevated levels and correlations with BMI, triglycerides, and HDL, with a proposed cut-off predicting PCOS with good sensitivity and specificity^7^. Consistent with this, the present study demonstrated higher ANGPTL-3 in PCOS and moderate predictive accuracy. The observed correlations with BMI, triglycerides, CRP, and HDL support a potential metabolic role; however, the underlying mechanisms remain incompletely understood. These associations suggest that inflammation, adiposity, and dyslipidemia may mediate the link between ANGPTL-3 and PCOS pathogenesis.

The relationship between ANGPTL-3 and MS has been examined in limited research. While some studies reported similar ANGPTL-3 levels between MS and controls, others found higher levels in obese subjects—particularly those with MS—and positive associations with BMI, blood pressure, total cholesterol, and LDL^17,18^. In a study by Aghasizadeh et al., genotypic frequencies of rs10789117 in the ANGPTL-3 were not associated with MS, while they were associated with a lower risk of MS in the control group^16^.

No prior studies have evaluated the predictive role of ANGPTL-3 for MS within PCOS populations. This study found higher ANGPTL-3 levels in PCOS patients with MS compared to those without MS and identified a cut-off value with moderate sensitivity and specificity. Unfortunately, no significant correlation was found between ANGPTL-3 levels and clinical or laboratory parameters in PCOS patients with MS.

The mechanism by which ANGPTL-3 may predict MS in PCOS remains unclear. ANGPTL-3 is known to act through lipid metabolism and insulin resistance under various conditions; these mechanisms alone may not fully explain the elevation of ANGPTL-3 in PCOS patients with MS, given the shared pathophysiological features of both conditions. Further studies exploring the association between ANGPTL-3 and metabolic disturbances could provide valuable insights. Previous research has shown that ANGPTL-3 influences arterial thickness and macrophage activity, both of which are linked to atherosclerotic plaque formation^19^. Additionally, elevated ANGPTL-3 levels have been associated with endothelial dysfunction^18^. These subclinical processes may help explain the lack of correlation observed between ANGPTL-3 and clinical parameters in our study. However, no mechanistic basis currently exists to suggest that ANGPTL-3 would selectively lose its metabolic associations specifically in the MS-positive state. Perhaps, this observed loss of significance is attributable to sample size constraints rather than true biological dissociation.

PCOS is increasingly recognized as a heterogeneous disorder encompassing distinct clinical, endocrine, and metabolic phenotypes. Longitudinal data indicate that MS risk and its progression vary across PCOS phenotypes, suggesting that metabolic burden is not uniformly distributed among affected women^1^. Although phenotype-based stratification was beyond the scope of the present study, the observed association between ANGPTL-3 and MS raises the hypothesis that this biomarker may preferentially reflect metabolically adverse PCOS subgroups rather than uniform endocrine characteristics across all phenotypes.

Recent studies have emphasized that dysglycemia and metabolic risk in PCOS are best predicted by combinations of anthropometric, metabolic, and endocrine parameters rather than single variables alone^20^. In this context, our findings do not propose ANGPTL-3 as a standalone diagnostic marker but rather position it as a complementary biomarker that may enhance metabolic risk stratification when integrated with established clinical and biochemical indicators.

The absence of correlations between ANGPTL-3 and metabolic parameters does not preclude its biological relevance. Emerging evidence suggests that hormonal and metabolic factors in PCOS interact in complex ways to influence downstream tissue responses and end organ sensitivity^21^. Accordingly, ANGPTL-3 may represent an integrative marker of cumulative metabolic burden rather than a direct mediator of isolated metabolic pathways, which may explain its discriminative capacity for MS despite weak correlations with individual components.

Differential diagnosis of menstrual disorders remains challenging, particularly in adolescents and young women, where hypothalamic–pituitary–ovarian axis immaturity may mimic features of PCOS^22^. Biomarkers capable of distinguishing PCOS accompanied by metabolic dysfunction from other causes of menstrual irregularity could therefore offer clinical value. In this regard, ANGPTL-3 may serve as an adjunctive tool to support metabolic risk assessment in PCOS rather than diagnostic classification alone.

The present study has several limitations. First, the cross-sectional design precludes causal inferences regarding the relationship between ANGPTL-3 and MS in PCOS. Second, the small sample size of MS-positive patients may have contributed to the absence of significant correlations between ANGPTL-3 and metabolic parameters; therefore, these findings should be interpreted with caution. Third, phenotype-based stratification of PCOS was not performed. Given the well-established heterogeneity of PCOS and the differential metabolic risk profiles across phenotypes, future studies incorporating phenotype-specific analyses are warranted to clarify whether ANGPTL-3 preferentially reflects metabolically adverse PCOS subgroups. Fourth, as all participants were of Turkish nationality, the generalizability of our findings to other ethnic populations may be limited. Finally, multiple testing may increase the risk of type I error, so the results should be interpreted with caution.

## CONCLUSION

In conclusion, this study demonstrates that ANGPTL-3 is markedly elevated in PCOS and is associated with the presence of MS. Although ANGPTL-3 showed moderate discriminative ability for MS, its role appears to reflect cumulative metabolic burden rather than direct linear associations with individual metabolic parameters. Taken together, our findings suggest that ANGPTL-3 may serve as a complementary biomarker to support metabolic risk assessment in PCOS. Future longitudinal and phenotype-based studies integrating ANGPTL-3 with established clinical and biochemical indicators are needed to further clarify its role in PCOS.

## Data Availability

The datasets generated and/or analyzed during the current study are available from the corresponding author upon reasonable request.
